# Retinoblastoma treatment in a Brazilian population. Presentation and long‐term results

**DOI:** 10.1002/cam4.6683

**Published:** 2024-01-19

**Authors:** Maria Teresa Brizzi Chizzotti Bonanomi, Maria Tereza A. de Almeida, Marianna A. Hollaender, Roberta Chizzotti Bonanomi, Mario Luiz Ribeiro Monteiro

**Affiliations:** ^1^ Division of Ophthalmology Hospital das Clínicas da Faculdade de Medicina da Universidade de São Paulo São Paulo Brazil; ^2^ Laboratory for Investigation in Ophthalmology (LIM‐33), Faculdade de Medicina FMUSP Universidade de São Paulo São Paulo Brazil; ^3^ ITACI (Treatment of Children with Cancer Institute) and Children's Institute Hospital das Clínicas da Faculdade de Medicina da Universidade de São Paulo São Paulo Brazil

**Keywords:** chemotherapy, eye enucleation, neoplasm staging, retinoblastoma, survival, treatment outcome, visual acuity

## Abstract

**Introduction:**

Retinoblastoma is a malignant tumor with a high cure potential when proper therapy is used. The purpose of this paper is to report the clinical features and outcomes of patients with retinoblastoma who were treated with a combination of local and systemic chemotherapy‐based protocols.

**Method:**

We retrospectively studied patients treated with systemic chemotherapy plus local treatment between 2003 and 2015 with a follow‐up ≥2 years. We correlated clinical and pathological characteristics with decimal visual acuity (VA) and death.

**Results:**

Among 119 patients, 60% had unilateral disease (UNI), and 52% were male. The median presentation age was 19.5 months, 10% had a positive family history, and the most frequent sign was leukocoria (68.8%). Advanced disease was more frequent in eyes with UNI (98.4%) than in eyes with bilateral retinoblastoma (BIL: 55.3%). Enucleation was performed in 97% of UNI eyes and in 55.8% of BIL eyes. The overall globe salvage was 26.6%, 44.25% of BIL eyes. Bilateral enucleation was required in 5%. High‐risk pathologic features occurred in 50% and 37% of eyes enucleated without and with neoadjuvant chemotherapy, respectively. High‐risk features were related to the presence of goniosynechiae in the pathologic specimen and were more frequent in children younger than 10 months or older than 40 months. Extraocular disease was present in 5% of patients, and the death rate related to metastasis of the tumor was 8%. The final VA was ≥ 0.7 in 72.8% and ≥0.1 in 91% of BIL patients.

**Conclusions:**

Treatment of retinoblastoma with conservative systemic‐based chemotherapy was associated with an excellent survival rate (92%). Albeit the low overall globe salvage rate, in BIL patients, approximately half the eyes were conserved, and a satisfactory functional visual result was achieved The evaluated protocol is an important treatment option, especially in developing countries.

## Graphical abstract

We report the clinical features and outcomes of patients with retinoblastoma treated with systemic chemotherapy and local consolidation with a follow‐up period of greater than 2 years in Brazil. We discuss the high‐risk features of this neoplasm and emphasize the high success rate obtained, including 92% survival and reading visual acuity in 72.8% of bilateral disease.

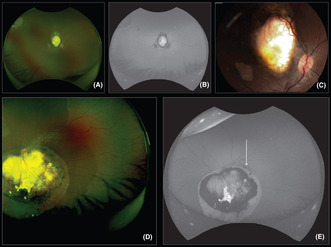

## 1 INTRODUCTION

Retinoblastoma is a malignant tumor with a high potential for cure when proper therapy is used. The origin of most cases of retinoblastoma is loss of the two alleles of the tumor suppressor gene RB1.^1^ With a reported incidence of one in 15,000–20,000 live births, there has been an estimated increase in incidence in European countries, likely due to improved survival, reproductive capability, and confidence in survival of hereditable retinoblastoma.^2^ In Brazil, based on data from the registry of the National Institute of Cancer, an incidence adjusted for the world population of 7.02 per million for children under 4 years of age has been estimated.^3^ Positive family history varies from 1% up to 18%, being more frequent in bilateral retinoblastoma,^4^, ^5^, ^6^ but only up to 6% of patients test positive for RB1 mutations.^1^ Children with intraocular retinoblastoma usually present with abnormalities in the red pupil reflex which turns white, a finding clearly visible on flash photographs. As the tumor grows, it exits the eye through the optic nerve, sclera, or emissary veins, causing proptosis. The prevalence of leukocoria and proptosis is reported to vary from 75% to 83% and 6% to 17%, respectively.^4^, ^5^, ^7^, ^8^

The prognosis of retinoblastoma is largely dependent on the classification of the intraocular tumor,^9^, ^10^ systemic staging^11^ and adequate management. Treatment has traditionally been based on enucleation of the affected eye or, when attempting to preserve the eye (especially in bilateral cases), external beam radiation therapy (EBRT). In the second half of the 1990s, a series of reports elucidated the treatment of this disease while attempting to avoid external beam radiotherapy (EBRT).^12^ The protocol, which consisted of drugs alone or in combination for chemoreduction of tumor volume plus local consolidation treatment,^13^, ^14^, ^15^ aiming to save lives and to maintain useful vision. More recently, intra‐arterial chemotherapy (IAC) was introduced and proposed as first‐line treatment for children with advanced unilateral or bilateral retinoblastomas.^16^, ^17^ However, IAC is still too costly and sophisticated to make it feasible on a large scale, especially in developing countries. By contrast, systemic chemotherapy for chemoreduction plus local consolidation treatment is currently a feasible option, even in low‐income populations, although it involves complex decisions based on tumor size, age, risk of metastasis, patient response, chemotherapeutic toxicity, and clinical expertise.

The purpose of this paper was to report the clinical and pathologic features and outcomes, (including functional results) of a large series of patients treated as a multidisciplinary approach with systemic chemotherapy plus local tumor consolidation managed by a single expert physician at the University of São Paulo Medical School, in São Paulo, Brazil.

## 2 METHODS

Data from the records of 130 patients treated between 2003 and 2015 were retrospectively reviewed. The study included data on some patients previously published,^18^ was approved by the Institutional Review Board Ethics Committee at our institution (*CAPPesq*, Approval Number: 1090/06) and was compliant with the principles of the Declaration of Helsinki.

We used the clinical grouping of the Los Angeles (Appendix S1) International intraocular retinoblastoma classification (IIRC),^9^ using fundus and slit lamp examination. All patients underwent brain MRI and ultrasound. Inclusion criteria were a minimal follow‐up of 2 years in the oncology center and of 5 years to determine fatal outcomes. Evidence of patient survival 5 years after the end of the treatment period obtained by any communication means was valid; otherwise, the patient was excluded from the survival calculation.

Treatment was based on systemic chemotherapy with two or three drugs (etoposide, vincristine, and carboplatin) coupled with an 810 nm laser or cryotherapy consolidation every 4 weeks under general anesthesia (Appendices S2 and S3).^9^, ^10^, ^18^, ^19^ Fundus evaluation before each section and treatment was performed by the same physician (MTBCB). Although tumor recurrence is better defined now, during the study period, an active disease after 3 cycles of etoposide, vincristine, and carboplatin (VEC) was considered recurrence if there was regrowth of treated retinal tumor, subretinal, or vitreous recurrence, or new tumor, the one not observed at initial diagnosis,^20^, ^21^ with 1, 2, or 3 months intervals of examination under sedation, in accordance with the fundus drawing. Detection of recurrence was treated with an alternative drug regimen, usually topotecan combined with irinotecan, before deciding on EBRT or enucleation. EBRT was used as a last resort for tumor recurrence before secondary enucleation. Patients with eventual recurrence or incomplete remission manageable with radioactive plaque were referred to another institution, and in more recent years, vitreous seeding was managed with 20 μg of intravitreous melphalan, in agreement with recent recommendations.^22^ Primary enucleation was indicated in all patients in unilateral retinoblastoma (UNI) group E or D without possibility of vision recovery, except for patients aged 1 year or younger since they might actually have bilateral disease that had not yet appeared in the second eye. UNI patients were treated with chemotherapy prior to enucleation only if there were technical difficulties with the surgery, such as orbital cellulitis with proptosis unresponsive to steroids; otherwise, they received only systemic treatment based on the high‐risk features (HRF) found in the pathology specimen, meaning retrolaminar nerve invasion, scleral invasion, and, after 2011, massive choroidal invasion.^23^ The treatment following HRF diagnosis was made with six sessions of VEC, and the above rules were also applied for exceptional cases of primary enucleation in bilateral retinoblastoma (BIL).

We analyzed the age at presentation (AP), laterality, family history (FH), presentation sign (PS), and visual acuity (VA) of the best‐seeing eye. PSs were divided into six categories: (1) leukocoria, (2) strabismus, (3) proptosis, (4) buphthalmia and phthisis, (5) routine familial examination, and (6) uveitis masquerade. Enucleated eyes were classified based on the 8th edition of the American Joint Committee on Cancer (AJCC) staging manual into pTNM or ypTNM if the patient had not or had neoadjuvant chemotherapy, respectively.^11^

The pathologic classification was analyzed in relation to AP intervals, FH, and PS. To understand the surgical indications and prevent enucleation of eyes potentially suitable for rescue, we analyzed the relationship between the IIRC or pTNM stages and the presence of iris neovascularization or goniosynechiae in pathology specimens.

The number of fatal outcomes was very low, posing difficulties in the statistical analysis. We studied the correlations of FH, AP, PS, IIRC, enucleation, and pathologic staging with the fatal outcome. Extraocular disease was considered based on pathology (pT4) and not on imaging. Aiming to address the vision of the better eye in BIL, the decimal VA was divided into four intervals: (A) 1.0–0.7, (B) 0.6–0.1, (C) lower than 0.1, and (D) no light perception (NLP) or bilateral enucleation.

### 2.1 Statistical analysis

Statistical analyses were performed using IBM SPSS Statistics version 17.0 (IBM Corp., Armonk, NY, USA). Qualitative variables were described using absolute and relative frequencies, and the association between the groups was verified using the chi‐square test or Fisher's exact test. Descriptive statistics of quantitative variables include mean values ± standard deviation (SD), median, maximum, and minimum values. The groups were compared using the Mann–Whitney test. The level of statistical significance was set at *p* ≤ 0.05.

## 3 RESULTS

Among the 130 registered patients, 11 were excluded: two had no tumor in the enucleated eye; six were registered in the hospital but treated elsewhere and three had no follow‐up after enucleation. Among the 119 included patients, the disease laterality was UNI in 71 (60%) and BIL in 48 (40%). FH, AP, sex, and PS are shown in Table 1. The follow‐up duration did not differ between the groups, and was ≥5 years, 3–4 years, and 2 years for 87%, 10%, and 3% of the UNI patients respectively, and 91.4%, 5.7%, and 2.8% of the BIL patients, respectively. Evidence of optic nerve invasion on MRI was present in 12 of 44 reliable images and was apparently associated with proptosis, buphthalmia, phthisis, and masquerade syndrome although the numbers were too small for a definitive analysis.

**TABLE 1:** Descriptive values of the 119 patient characteristics in both retinoblastoma laterality groups. The BIL group had more frequent FH positivity and an earlier age of presentation.

Variable	48 BIL: *n* (%)	71 UNI: *n* (%)	Total 119	*p*
FH^a^				
N	32 (76.2)	57 (91.9)	89 (85.5)	0.025^c^
Y	10 (23.8)	5 (8.1)	15 (14.4)	
Age in months				
MD ± PD	15.27 ± 13.28	28.57 ± 16.18	23.07 ± 16.3	<0.001^d^
Median	13.00	29.50	19.5	
(interval)	(1 day–82 months)	(2.6–72 months)		
Sex				
Male	24 (50)	38 (53.5)	62 (52.1)	0.706^c^
Female	24 (50)	33 (46.5)		
PS^b^				
1: leukocoria	32 (69.6)	45 (68.2)	77 (68.8)	0.151^e^
2: strabismus	5 (10.9)	6 (9.1)	11 (9.8)	
3: proptosis	1 (2.2)	7 (10.6)	8 (7.1)	
4: buphthalmos/phthisis	3 (6.5)	4 (6.1)	7 (6.3)	
5: routine checkup	5 (10.9)	1 (1.5)	6 (5.4)	
6: masquerade	0 (0)	2 (3)	2 (1.8)	
1 plus 3	0 (0)	1 (1.5)	1 (0.9	

Abbreviations: BIL, bilateral; FH, family history; M ± PD, mean ± pattern deviation; mo, month; N, no; n, number; PS, presentation sing; UNI, unilateral; Y, yes.

^a^ 104 patients.

^b^ 112 patients.

^c^ Chi‐square test.

^d^ Mann–Whitney nonparametric test.

^e^ Fisher's exact test.

The IICR, available for 158 eyes (111 patients) was as follows: Group A: 7 (4.4%), including 0 UNI and 7 BIL; group B, 28 (17.7%), including 0 UNI and 28 BIL; group C, 8 (5%), including 1 UNI and 7 BIL; group D, 54 (34.1%), including 23 UNI and 31 BIL; and group E, 61 (38.7%), including 40 UNI and 21 BIL. The regrouping of less advanced (A, B, or C) and more advanced retinoblastoma (D, E) is shown in Table 2. Advanced retinoblastoma at presentation was significantly more frequent in eyes with UNI, whereas less advanced disease prevailed in eyes with BIL (*p* < 0.001).

**TABLE 2:** International group classification of 158 eyes (111 patients).

Regrouping	UNI: *n* (%)	BIL: *n* (%)	Total (eyes)
A/B/C	1 (1.56)	42 (44.68)	43
D/E	63 (98.44)	52 (55.32)	115
TOTAL	64	94	158

Abbreviations: BIL, bilateral disease; n, number; UNI, unilateral disease.

*Note*: Advanced groups of retinoblastomas at presentation were more frequent in UNI eyes, while less advanced groups were more frequent in BIL eyes (*p* < 0.001, chi‐square test). Regrouping the less advanced intraocular disease, in short: group A: small tumor; B: larger tumors confined to the retina; C: associated minimal fluid and/or seeds, plus more advanced disease; D: massive subretinal or vitreous seeds; E: globe alterations secondary to the tumor.

The total number of enucleations is shown in Table 3:. Groups UNI and BIL differed in the number of enucleations, the overall globe salvage was 26.6%, 44.25% of BIL eyes. Primary enucleation was more frequent in UNI patients. Two eyes (2.8%) in UNI patients and 42 eyes (44.2%) in BIL patients were treated conservatively. Bilateral enucleation was performed in six children (5% of the total and 12.5% of BIL patients).

**TABLE 3:** Enucleation in UNI and BIL patients (one eye missing in each group).

Enucleation	UNI *n* (%)	BIL *n* (%)
Yes	68/70 (97.1)	53/95 (55.8)
Yes (after chemo ‐included in the anterior)	9/68 (13.2)	50/53 (94.3)
No	2 (2.8)	42 (44.2)

Abbreviations: BIL, bilateral disease; UNI, unilateral disease; n, number.

*Note*: Under “Yes” is the total number of enucleation including those performed after chemotherapy.

Regarding the pathology specimens of primary enucleated eyes, the classification of the pTNM stages for UNI and BIL eyes was as follows. Among the 56 UNI eyes with available registry data, the distribution of classification was pT1: 4 (7.1%); pT2a: 19 (33.9%); pT2b: 5 (8.9%); pT3a: 5 (8.9%); pT3b: 12 (21.4%); pT3c: 9 (16.1%); and pT4: 2 (3.6%). For 3 BIL eyes, two eyes were classified as pT1 (66.7%), and one eye was classified as pT2a (33.3%). Therefore, HRF were present in approximately 50% of UNI but in no BIL eyes. When only UNI patients were included, there was no correlation between pTNM stage and HF or PS, but some correlation with AP (Table 4). The total number of BIL eyes with pTNM classification was too small for analysis. The data of 54 eyes enucleated after chemotherapy (9 UNI and 45 BIL) were analyzed, and the distribution by subgroup was as follows: ypT0: 1 (1.8%); ypT1: 25 (46.29%); ypT2a: 5 (9.2%); ypT2b: 3 (5.5%); ypT3a: 6 (11.1%); ypT3b: 3 (5.5%); ypT3c: 6 (11.1%); ypT3d: 1 (1.8%); and ypT4: 4 (7.4%). Most of them, 34 (63%), lacked HRF. When only BIL patients were included, there was no correlation between ypTNM stage and AP, HF or PS. The total number of UNI eyes with ypTNM classification was too small for analysis. Therefore, pathologically proven extraocular disease (pT4) was present in six (5%) patients (four UNI and two BIL).

**TABLE 4:** Correlation between AP and pTNM classification in 54 eligible enucleated eyes of UNI. Some age groups showed significant differences in pathologic stage (numbers in italics). AP < 10 months was most often at stages pT2b and pT3b, AP 10–40 months at stage pT2a, and AP > 40 months at 1 stage pT3b. Fisher's exact test (*p* = 0.022).

pTNM	AP				
	< 10 m	10–20 m	20–30 m	30–40 m	> = 40 m
pT1	0 (0.0)	3 (25.0)	0 (0.0)	0 (0.0)	1 (8.3)
pT2a	0 (0.0)	*5 (41.7)*	*5 (45.4)*	*7 (50.0)*	2 (16.7)
pT2b	*2 (40.0)*	1 (8.3)	1 (9.1)	1 (7.2)	0 (0.0)
pT3a	1 (20.0)	1 (8.3)	0 (0.0)	1 (7.2)	2 (16.7)
pT3b	*2 (40.0)*	2 (16.7)	1 (9.1)	1 (7.2)	*6 (50.0)*
pT3c	0 (0.0)	0 (0.0)	4 (36.4)	3 (21.2)	1 (8.3)
pT4	0 (0.0)	0 (0.0)	0 (0.0)	1 (7.2)	0 (0.0)

Abbreviations: AP, age of presentation; m, months; UNI, unilateral disease, (%).

*Note*: Pathologic (pTNM) classification: pT1: Intraocular tumor(s) without any local invasion, focal choroidal invasion, or pre‐ or intra‐laminar involvement of the optic nerve head; pT2: intraocular tumor(s) with local invasion; pT2a: concomitant focal choroidal invasion and pre‐ or intra‐laminar involvement of the optic nerve head; pT2b: tumor invasion of the stroma of the iris and/or trabecular meshwork and/or Schlemm's canal; pT3: intraocular tumor(s) with significant local invasion; pT3a: massive choroidal invasion (>3 mm in largest diameter, or multiple foci of focal choroidal involvement totaling >3 mm, or any full‐thickness choroidal involvement); pT3b: retrolaminar invasion of the optic nerve head, not involving the transected end of the optic nerve; pT3c: any partial‐thickness involvement of the sclera within the inner two‐thirds; pT3d: full‐thickness invasion into the outer third of the sclera and/or invasion into or around emissary channels; and pT4: evidence of extraocular tumor: tumor at the transected end of the optic nerve, tumor in the meningeal spaces around the optic nerve, or full‐thickness invasion of the sclera with invasion of the episclera, adjacent adipose tissue, extraocular muscle, bone, conjunctiva, or eyelids.

Notably, 31 primary enucleated eyes had no HRF based on pathologic specimens, indicating a stage of pT2b or less. Five eyes in clinical group D or E were classified as pT2b; thus, pathologic anterior segment invasion indicated enucleation. For the remaining 26 eyes with stages below pT2b, clinical groups C or D and E represented 4% and 96%, respectively. In this set of eyes, iris neovascularization was present in 57.7% and goniosynechiae in 46.2%. Considering all eligible anatomic data, iris neovascularization was present in 76% of eyes and had no relationship with pTNM stages. Goniosynechiae was present in 70% of the eyes and was more prevalent in group pT3b eyes (Fisher's exact test *p* = 0.0406).

Of the 111 patients in the entire cohort, nine (8%) died following metastasis of the tumor. In the UNI group, among 65 children whose data were compiled, six (9.2%) died. All deaths were of patients with advanced disease, one in group D and five in group E. All but one case involved enucleation; one had and four had not undergone neoadjuvant chemotherapy, and two had received EBRT. We found no studied variable related to death (PA with nonparametric Mann–Whitney *U* test; FH, PS, enucleation and pTNM or ypTNM with Fisher's exact test). In the BIL group, three of 43 (6.5%) patients died. All deaths occurred with advanced IIRC of the worse eye: two (66.77%) in group D and one (33.3%) in group E. All had received neoadjuvant chemotherapy, and one also had EBRT. None of the studied variables was related to death (PA with nonparametric Mann–Whitney *U* test; FH, PS, enucleation, and pTNM or ypTNM with Fisher's exact test). From the eyes enucleated from patients who died, all but two had post laminar invasion (pT3 or ypT3 and over). In the UNI group one eye was pT2a and one had no registry.

**FIGURE 1:**
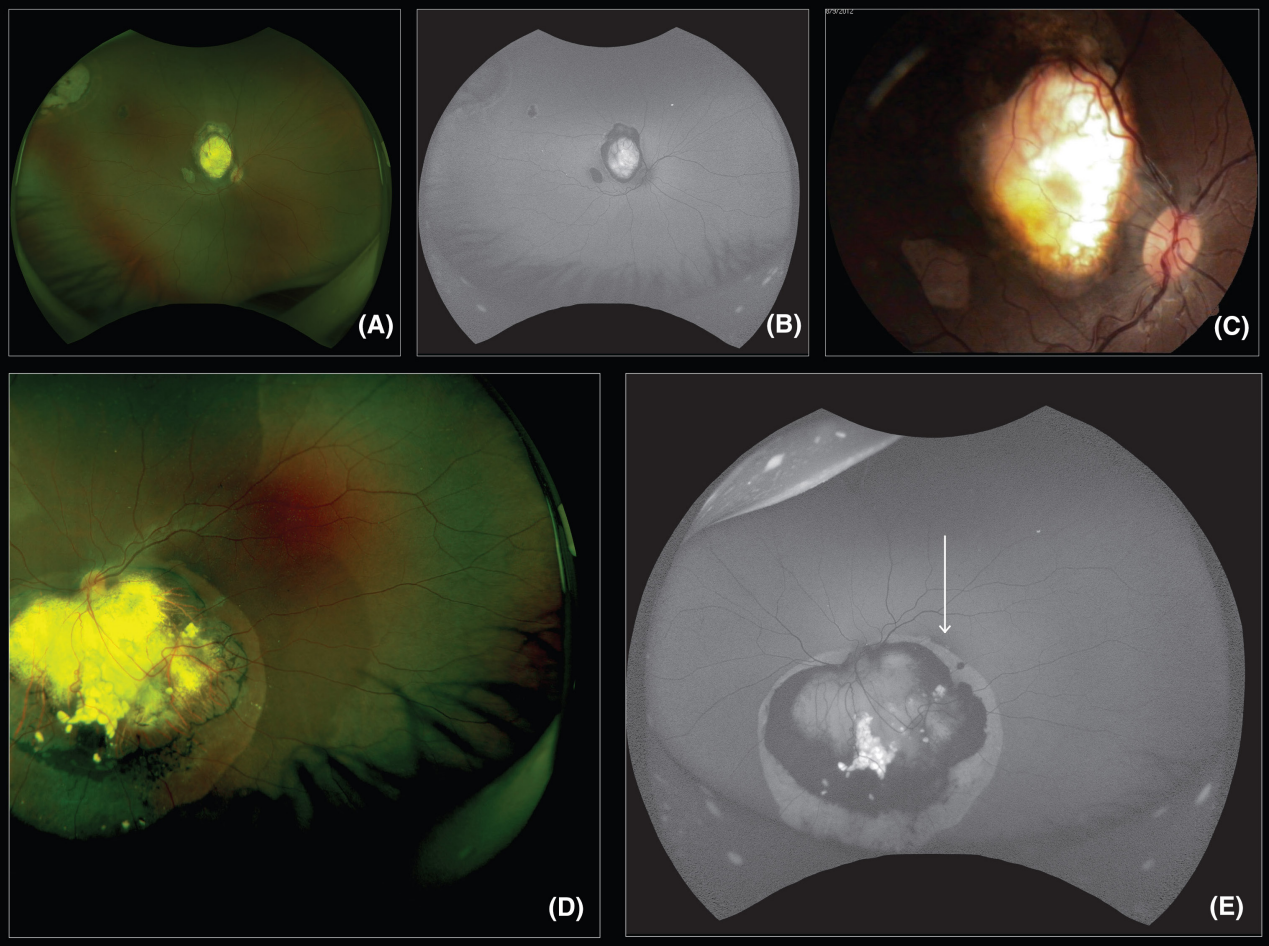
Right (only) eye of a bilateral retinoblastoma with final vision 0.5, 15 years after treatment: (A) pseudocolor, (B) autofluorescence, and (C) retinography showing that almost all xanthophyl pigment was over a bare sclera atrophic area. Left (only) eye of another girl with final vision of 1.0, 18 years after treatment: (D) pseudocolor and (E) autofluorescence showing a small portion of the macula with normal pigmentation (arrow). Both patients were treated with systemic chemotherapy and TTT with foveal sparing.

VA data were available for 33 patients in the BIL group, and all patients with follow‐up of more than 2 years were already able to provide visual information, as shown in Table 5. A VA of 0.7 or better was more frequent but not exclusively in the absence of a macular tumor (Figure 1). Additionally, the presence of a macular tumor was more likely to result in enucleation of the second eye (*p* < 0.001).

**TABLE 5:** Visual acuity in the best‐seeing eye of 33 treated bilateral retinoblastoma patients.

VA interval	Macular tumor				Total
	No (*n* = 31)		Yes (*n* = 8)		
	*n*	%	*n*	%	
A: 1–0.7	23	74.2*	1	12.5	24 (72.7%)
B: 0.1–0.6	3	9.7	3	37.5	6
C: <0.1	3	9.7	0	0.0	3
D: enucleation	2	6.5*	4	50.0	6

Abbreviation: VA, visual acuity.

*Note*: Significant findings (*) were better VA in the absence of a macular tumor (interval A) and more likely enucleation of the second eye (interval D) in the presence of a macular tumor in the initial examination (*p* = 0.001, Fisher's exact test). The table includes six patients with bilateral enucleation (D). We excluded three without registry (one in interval C, two in interval D), three due to lack of follow‐up and three in the death group.

## 4 DISCUSSION

Across the large number of studies published on retinoblastoma, the difference in presentation and outcomes based on the population studied, that is, the country where the children live, is always striking. Brazil is a highly heterogeneous country in terms of income per capita, ranging from US$115 to US$457 in different regions (https://www.ibge.gov.br/). Although our hospital is in one of the most developed zones, many of our patients are from distant and less developed regions, which explains many of the late presentations. Although the main progress in retinoblastoma treatment in the past 20 years has consisted of local treatment through endoarterial chemotherapy,^16^ the fact that systemic treatment is a major development in the management of this disease^9^, ^10^ and available for many patients worldwide^24^, ^25^, ^26^, ^27^ highlights the importance of reporting our data.

In our study, the UNI frequency of 60%, median AP of 19.5 months, positive FH of 10%, proptosis as PS rate of 7%, and rate of patients in IIRC “D” or “E” of approximately 60% are similar to those in many studies.^4^, ^5^, ^6^, ^7^, ^28^, ^29^, ^30^, ^31^ Leukocoria is a major sign for the diagnosis of retinoblastoma and was the presentation of approximately 70% of our patients. The white, yellow, or pinkish color behind the lens occurs in many other conditions, congenital, or acquired. These conditions may simulate the tumor, must be differentiated from it for therapeutic purposes, and are known as pseudoretinoblastoma. The differential diagnosis may be challenging. In a large series of 604 such cases among 2775 patients referred as possible retinoblastomas, the most common diseases were Coats' disease, persistent fetal vasculature and, in older children, toxocara granuloma.^32^ These findings and the fact that the differential is related to the presentation age were confirmed by others.^33^

The total enucleation rate of 72% was also comparable to that in multicenter studies,^28^ but our overall globe salvage for this period is far below that reported worldwide.^4^, ^5^, ^7^, ^28^ The reason is certainly related to the fact they presented with advanced disease particularly due to the socioeconomic status of most of our population. Furthermore, intra‐arterial chemotherapy was available only in recent years at our institution, and our purpose was exactly to report data collected in patients treated solely with the current “Brazilian Protocol” for that period, based on systemic chemotherapy (Appendices S2 and S3). Our death rate was 8%, approximately the same as that in other Latin American countries.^34^, ^35^

A multicenter study by the AJCC on treatment success and global salvage reported the outcomes of 2854 eyes of 2097 patients.^25^ The AP was 17 months, and the IIRC was nearly 70% in groups D and E. The rate of enucleation was 55%, as performed in 41% as the primary surgery and 14% after failure of local treatment. The calculated hazard ratios for failure (EBRT or enucleation) for groups B, C, D, and E were 17.9, 29, 102.9, and 153.4, respectively. The AJCC study also evaluated metastasis‐related death and reported the results of 2085 patients in a multicenter study including 18 ophthalmic oncology centers in 13 countries, with follow‐up durations of 5 and 10 years.^26^ The IIRC in, groups A, B, C, D, and E was 6.1%, 16.5%, 7.3%, 38.8%, and 31.3%, respectively. Of these, 1397 eyes were treated with primary enucleation (48.1%), including 44 that underwent bilateral surgery. Bilateral enucleation was performed in six children in our study. An interesting finding in our study is that the presence of macular tumors at initial examination made the child more prone to undergo enucleation of the other eye (*p* = 0.001, Table 5). The authors reported that 109 patients developed metastasis and died, among whom 69 (5.1%) had already undergone enucleation. The median time from diagnosis to metastasis was 9.5 months. The authors also confirmed that for eyes at an advanced pTNM stage, the patients were more likely to have died due to metastasis at the 5‐year follow‐up. The cumulative survival rate decreased drastically from pT1 (99%) to pT4 (48%). Stage pT3d (external scleral or emissary vessel invasion) or pT4 (extraocular tumor) enucleated eyes had a 50‐fold and 77‐fold risk of metastasis relative to pT1, and thus these stages should indicate prompt systemic screening, as was the management for more than 5% of eyes with pT4 in our study.^26^

A global survey was conducted in patients who presented with the disease in 2017 and was estimated to have included more than half of all retinoblastoma cases worldwide in that year, including those from our center. Overall, the global data on upper‐middle income countries (UMICs) are very similar to our cases. The report included 4251 new patients from 153 countries.^24^ Of the entire cohort, 45.4% of patients were female, 30.8% had bilateral disease, and only 4.7% had a positive FH (8.4% for UMICs). A total of 3685 (84.7%) patients were from low‐ and lower‐middle income countries (LICs, LMICs). The most frequent PS was leukocoria (62.8%), followed by strabismus (10.25%) and proptosis (7.45%). Patients in high‐income countries (HICs) were diagnosed at a median age of 14.1 months, with 98.5% having intraocular retinoblastoma and 0.3% having metastasis. Patients from LICs were diagnosed at a median age of 30.5 months, with 49.1% having intraocular retinoblastoma and 18.9% having metastasis. The majority of patients had access to diagnostic imaging, enucleation, histopathology service, and intravenous chemotherapy.^24^

The follow‐up of the patients, including 4064 children in the global study was published in 2022.^27^ Extraocular tumors at presentation were reported in 0.8% of children from HICs, in 5.4% from UMICs, in 19.7% from LMICs, and in 42.9% from LICs. Enucleation or, rarely, exenteration was performed in 66.2%, similar to our total enucleation rate of 70%. Death was reported in 12.8% of the patients, and the 3‐year estimated survival rate was 99.5% for HICs, 91.2% for UMICs, 80.3% for LMICs, and 57.3% for LICs. Among our patients, death occurred only among the children who presented clinically as belonging to group D or E. We had an 8% death rate in the 5‐year follow‐up period (9% among UNI and 6.5% among BIL cases), which is equivalent to the estimated 3‐year survival for UMICs, in which mortality was reported to be related to income level, extraocular tumor, and older age at presentation among children up to 3 years.^27^ Mortality may be as high as 14% for advanced disease, despite all adjunctive interventions.^7^, ^30^, ^31^ We adhered to a low threshold when performing enucleation, so this may be the reason we had a relatively low death rate, which was approximately the same as that in Argentinian,^34^ Mexican,^35^ and Asian Indian cohorts^36^ but far higher than that in other studies.^6^, ^7^, ^29^

Most enucleated eyes in our study had low‐stage pathologic classification, and the presence of HRFs dictated post enucleation chemotherapy. The number of eyes of patients who had undergone neoadjuvant chemotherapy that had HRFs was lower than that of patients with primarily enucleated eyes, at 50% and 63%, respectively, similar to the findings Zhao et al. (*p* = 0.01).^30^ These authors state that chemotherapy before surgery could compromise survival by “downstaging” the disease, that more than 3 cycles of chemotherapy may delay surgery and compromise survival (*p* = 0.025) and that enucleation longer than 3.5 months for group D (*p* = 0.018) and 2 months for group E (*p* = 0.017) after diagnosis also decreases survival.

This issue was addressed by another group that found the same prevalence of these features in both sets of eyes (32% vs. 21%).^37^ However, they found a different pattern of invasion in the two groups. Primary enucleated eyes were significantly more likely to have massive choroidal invasion and postlaminar nerve invasion, while in the secondary enucleation group, ciliary body, and scleral invasion were the most common. The authors stated that systemic therapy may have preferentially targeted the choroid and optic nerve due to their complex vascular networks and that recurrence could preferentially involve the anterior uveal tract and the sclera.

The fact that many enucleated eyes had no HRFs in the pathologic specimens prompted concerns among our group of excessive aggressiveness for enucleation. All patients were in groups D or E, and most had anterior segment pathology. HRFs were more frequent in the age groups <10 months and > 40 months (Table 1). In five patients, enucleation was indicated based only on anterior segment invasion, which is now considered a manageable feature and seems not to be linked to recurrence, especially if isolated.^26^ It is possible that in these patients, surgery could be avoided.^23^ Otherwise, postlaminar optic nerve invasion (stage pT3b) was related to the presence of goniosynechiae, supporting the indication of enucleation in these cases. In fact, it seems that many retinoblastomas cause ischemia and neovascularization before invading the optic nerve or choroid. Anterior segment neovascularization marks an important trend in approaching the disease. In the prospective study of HRFs conducted by the Children's Oncology Group, iris neovascularization, and neovascular glaucoma were clinically associated with the presence of HRFs (69% vs. 37%), supporting the clinical use of anterior segment neovascularization as an indicator of possible HRFs.^38^

Concerning the functional result for the better eye in the BIL group, VA ≥0.7 was achieved in 72.8% of the patients, including patients in whom the better eye had a macular tumor (Figure 1). The largest cohort concerning vision included 140 eyes treated with chemoreduction and reported vision of ≥6/60 in 71% and ≥6/12 in 37% of nonenucleated eyes. A greater number of retinal tumors and the absence of foveolar involvement were predictors of a long‐term visual outcome of 6/12 or better.^39^ Our data considered only the better eyes, which could explain the better outcomes than the previous cohort. The tumor may completely involve the macula in up to 50% of cases.^39^, ^40^ In a report of 10 years of experience, the VA of the best eye with respect to normal vision was 26% in eyes with macular retinoblastoma and 90% in eyes without it.^41^ In our patients, the influence of macular tumors on the visual outcome was significant only for VA ≥0.7 (*p* = 0.001). Patching should be considered in these patients.^42^ Often, we attempt to treat central tumors with macular‐sparing lasers, but we have to be very cautious with the technique. In a study of only more advanced disease, in 32 group D eyes of 27 patients, transpupillary thermotherapy was the only variable linked to worse vision by multivariate analysis.^8^

## 5 CONCLUSION

In conclusion, treating retinoblastoma with a conservative systemic‐based chemotherapy is associated with an excellent 92% survival rate. Albeit the low overall globe salvage rate, in BIL patients, approximately half of the eyes could be conserved, and a satisfactory functional visual result was achieved. The evaluated protocol is an important treatment option particularly in developing countries.

## AUTHOR CONTRIBUTIONS

**Maria Teresa Brizzi Chizzotti Bonanomi:** Conceptualization (lead); data curation (supporting); formal analysis (equal); investigation (lead); methodology (lead); project administration (lead); supervision (lead); validation (equal); visualization (equal); writing – original draft (lead); writing – review and editing (equal). **Maria Tereza Assis de Almeida:** Conceptualization (equal); data curation (equal); methodology (equal); visualization (equal). **Marianna A. Hollaender:** Data curation (lead); software (equal); validation (equal); visualization (equal); writing – original draft (supporting); writing – review and editing (supporting). **Roberta Chizzotti Bonanomi:** Data curation (lead); software (equal); validation (equal); visualization (equal); writing – original draft (supporting); writing – review and editing (supporting). **Mario Luiz Ribeiro Monteiro:** Validation (equal); visualization (equal); writing – original draft (equal); writing – review and editing (lead).

## ETHICS STATEMENT

The study was approved by the local ethics committee and complied with the principles of the Declaration of Helsinki.

## Supporting information


Appendix S1

Appendix S2

Appendix S3

## Data Availability

Due to the nature of the research and ethical restrictions, supporting data are not available because the participants of this study did not give written consent for their data to be shared publicly. Data from two patients are available upon direct request from the corresponding authors.
