# Supplementary material for: Retinoblastoma treatment in a Brazilian population. Presentation and long‐term results
Source: Cancer Med. 2024 Jan 19;13(3):e6683. doi: 10.1002/cam4.6683 (PMC10905530; doi:10.1002/cam4.6683)
Supplement: Supplementary file 1 — Appendix S1 [file CAM4-13-e6683-s003.docx]

SUPPLEMENT 1 International intraocular retinoblastoma classification, based on reference (9)

|  |  |  |
| --- | --- | --- |
| Group A (very low risk) | All tumors are 3mm or smaller, confined to the retina, and located at least 3mm from the fovea and 1.5mm from the optic nerve. No vitreous or subretinal seeding is allowed |  |
| Group B (low risk) | Retinal tumors may be of any size or location, not in group A. No vitreous or subretinal seeding allowed. A small cuff of subretinal fluid extending no more than 5mm from the base of the tumor is allowed |  |
| Group C (moderate risk) | Any seeding must be local, fine and limited so as to be theoretically treatable with a radioactive plaque. Retinal tumors are discrete and of any size and location. Up to one quadrant of subretinal fluid may be present |  |
| Group D (high risk) | Eyes with more extensive seeding than group C. Massive and /or diffuse intraocular disseminated disease may consist of fine or “greasy” vitreous seeding or avascular masses. Subretinal seeding may be plaque-like. Includes exophytic disease and more than one quadrant of retinal detachment |  |
| Group E (very high risk) | Eyes with one or more of the following: irreversible neovascular glaucoma, massive intraocular hemorrhage, aseptic orbital cellulitis, tumor anterior to the anterior vitreous face, tumor touching the lens, diffuse infiltrating retinoblastoma, phthisis or pre-phthisis. |  |
