# Supplementary material for: Retinoblastoma treatment in a Brazilian population. Presentation and long‐term results
Source: Cancer Med. 2024 Jan 19;13(3):e6683. doi: 10.1002/cam4.6683 (PMC10905530; doi:10.1002/cam4.6683)
Supplement: Supplementary file 2 — Appendix S2 [file CAM4-13-e6683-s002.zip › Appendix S2.docx]

Appendix S2: Outline of the protocol for the retinoblastoma treatment in the period of the study.
