# Supplementary material for: Retinoblastoma treatment in a Brazilian population. Presentation and long‐term results
Source: Cancer Med. 2024 Jan 19;13(3):e6683. doi: 10.1002/cam4.6683 (PMC10905530; doi:10.1002/cam4.6683)
Supplement: Supplementary file 2 — Appendix S2 [file CAM4-13-e6683-s002.zip › cam46683-sup-0002-AppendixS2.pdf]

# RETINOBLASTOMA - BRAZILIAN PROTOCOL

## Management scheme for unilateral, unifocal intraocular tumors

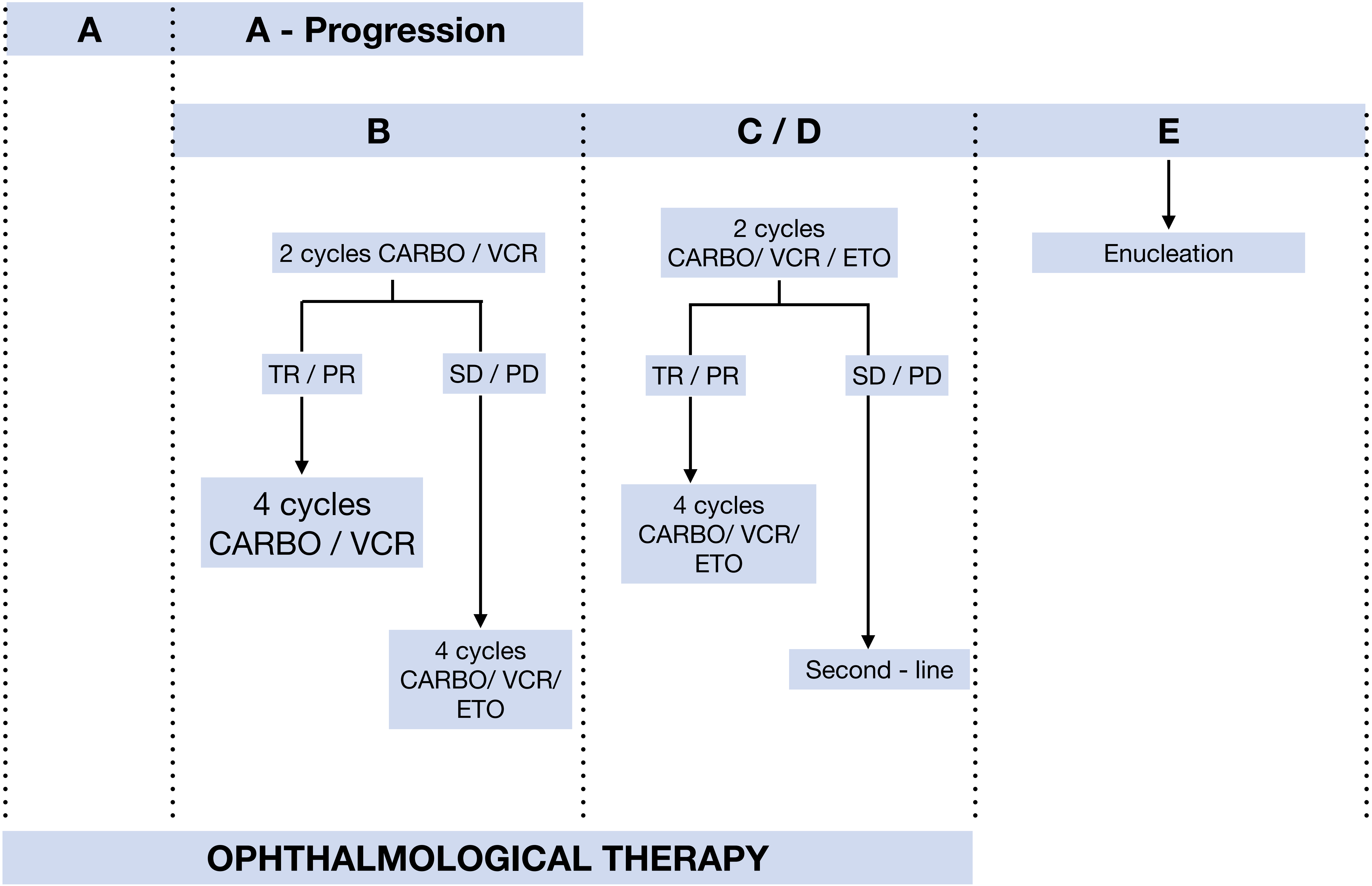


**(CARBO) carboplatin; (VCR) vincristine; (ETO) etoposide; (TR) total response; (PR) partial response; (SD) Stable disease; (PD) progressive disease**
