# Supplementary material for: Retinoblastoma treatment in a Brazilian population. Presentation and long‐term results
Source: Cancer Med. 2024 Jan 19;13(3):e6683. doi: 10.1002/cam4.6683 (PMC10905530; doi:10.1002/cam4.6683)
Supplement: Supplementary file 3 — Appendix S3 [file CAM4-13-e6683-s001.zip › Appendix S3.docx]

Appendix S3: Chemotherapy protocol: drug administration and cumulative dosage.
