# Supplementary material for: Retinoblastoma treatment in a Brazilian population. Presentation and long‐term results
Source: Cancer Med. 2024 Jan 19;13(3):e6683. doi: 10.1002/cam4.6683 (PMC10905530; doi:10.1002/cam4.6683)
Supplement: Supplementary file 3 — Appendix S3 [file CAM4-13-e6683-s001.zip › cam46683-sup-0003-AppendixS3.pdf]

Chemotherapy Protocol

	Dose (mg/m2)	D1	D2
Carboplatin	540	X	
Vincristine	1.5	X	
Etoposide	150	X	X

Cumulative Dose	4 cycles	6 cycles
Carboplatin	-	3240
Etoposide	1200	1800
